# A novel platelet risk score for stratifing the tumor immunophenotypes, treatment responses and prognosis in bladder carcinoma: results from real-world cohorts

**DOI:** 10.3389/fphar.2023.1187700

**Published:** 2023-05-04

**Authors:** Dingshan Deng, Xiaowen Li, Tiezheng Qi, Yuanqing Dai, Neng Liu, Huihuang Li

**Affiliations:** ^1^ Department of Urology, National Clinical Research Center for Geriatric Disorders, Xiangya Hospital, Central South University, Changsha, Hunan, China; ^2^ National Clinical Research Center for Geriatric Disorders, Xiangya Hospital, Central South University, Changsha, Hunan, China; ^3^ Xiangya School of Medicine, Central South University, Changsha, Hunan, China; ^4^ Teaching and Research Section of Clinical Nursing, Xiangya Hospital, Central South University, Changsha, Hunan, China

**Keywords:** platelet, bladder carcinoma, immunotherapy, prognosis, tumor microenvironment

## Abstract

**Background:** Although the durable efficacy of immune checkpoint inhibitors (ICIs) in BLCA has been confirmed in numerous studies, not all patients benefit from their application in the clinic. Platelets are increasingly being found to be closely associated with cancer progression and metastasis; however, their comprehensive role in BLCA remains unclear.

**Methods:** We comprehensively explored platelet expression patterns in BLCA patients using an integrated set of 244 related genes. Correlations between these platelet patterns with tumor microenvironment (TME) subtypes, immune characteristics and immunotherapy efficacies were explored. In addition, a platelet risk score (PRS) was generated for individual prognosis and verified the ability to predict prognosis, precise TME phenotypes, and immunotherapy efficacies.

**Results:** Genes were clustered into two patterns that represented different TME phenotypes and had the ability to predict immunotherapy efficacy. We constructed a PRS that could predict individual prognosis with satisfactory accuracy using TCGA-BLCA. The results remained consistent when PRS was validated in the GSE32894 and Xiangya cohort. Moreover, we found that our PRS was positively related to tumor-infiltrating lymphocytes (TILs) in the TCGA-BLCA and Xiangya cohort. As expected, patients with higher PRS exhibited more sensitive to immunotherapy than patients with lower PRS. Finally, we discovered that a high PRS indicated a basal subtype of BLCA, whereas a low PRS indicated a luminal subtype.

**Conclusion:** Platelet-related genes could predict TME phenotypes in BLCA. We constructed a PRS that could predict the TME, prognosis, immunotherapy efficacy, and molecular subtypes in BLCA.

## Introduction

Bladder cancer (BLCA) is considered as the 10th commonest malignancy all over the world, leading to approximately 210,000 deaths worldwide annually (Sung et al., 2021). Currently, invasive cystoscopy and tissue biopsy have become the gold standard for BLCA detection and surveillance (Babjuk et al., 2022), and some novel methods show bright prospects, such as urine DNA methylation and urine exosomes assay (Chen et al., 2020; Wei et al., 2022). As for treatment, although neoadjuvant chemotherapy has become the standard of care, the prognoses of advanced BLCAs are still not satisfied due to the insensitivity to this therapy (Patel et al., 2020). Fortunately, urologists found great immunogenicity such as a high tumor mutation burden (TMB) in the BLCA (Patel et al., 2020), which could be coped with immunotherapy, especially including immune checkpoint inhibitors (ICIs) which was considered to have great clinical effect for advanced BLCA (Sharma et al., 2017; Necchi et al., 2018). However, not every patient is sensitive to treatment with ICIs (Bagchi et al., 2021). Therefore, predicting patient response to ICIs is of great significance. The tumor microenvironment (TME) denotes the cancer cells, non-cancerous cells, and their corresponding secreted factors in the tumor (Xiao and Yu, 2021). It can be divided into binary groups according to the appearance of tumor-infiltrating lymphocytes (TILs) (Chen and Mellman, 2017). Inflamed tumors have a high level of TIL infiltration and generally express cytokines that activate immunity, whereas non-inflamed tumors have the opposite characteristics (Chen and Mellman, 2017). Theoretically, ICIs re-invigorated tumor-cytotoxic T cells to inhibit tumor growth in the TME (Duan et al., 2020), thus indicating better efficacy in inflamed tumors. For tumors with limited TILs, patients showed a significantly lower response to ICI treatment (Beer et al., 2017; Quail and Joyce, 2017), while inflamed tumors such as melanoma and renal cell carcinoma show desirable clinical response (Tumeh et al., 2014; McDermott et al., 2018). Thus, discriminating these two phenotypes may contribute to predicting the efficacy of ICIs; in addition, the transformation of non-inflamed tumors into inflamed ones can improve ICI efficacy (Duan et al., 2020).

Platelets, which play a crucial role in coagulation and hemostasis after a mechanical injury to the vasculature, are closely related to cancer progression and metastasis (Schlesinger, 2018). Riess first noted that thrombocytosis was associated with solid tumors over a century ago (Riess, 1872). With the progress of modern biotechnology, recent studies have found that platelets influence TME, cancer progression and metastasis in various ways. Platelets envelop tumor cells by binding to mucins on the surface of cancer cells, which interfere with NK cells’ recognition of tumor cells and physically protect them from NK cells’ attacks (Goubran et al., 2013). Activated platelets can release VEGFA, PDGF and other pro-angiogenic factors to promote blood vessel formation in TME, thereby promoting tumor growth (De Palma et al., 2017). In addition, platelets can produce TGF-β to inhibit the production of IFN-γ, T cell proliferation and promote tumor cell metastasis (Haemmerle et al., 2018). Thus, platelet-related genes may play key roles in cancer progression and immunity (Haemmerle et al., 2018). Von Willebrand factor (VWF), which plays a vital part in platelet adhesion along the endothelium, is involved in cancer metastasis and inflammation (Patmore et al., 2020). Furthermore, protein tyrosine phosphatase non-receptor type 6 (PTPN6) was found to be a negative regulator of the inflammatory cell death pathways that take part in cancer immunity (Speir et al., 2020). In lung tumor model, peroxiredoxin 6 (PRDX6) contributes to tumor growth, which is associated with JAK2/STAT3 pathway (Yun et al., 2015). However, the comprehensive role of platelet-related genes need to be further explored. In this study, we reported that platelets promote an inflamed TME, and constructed a platelet risk score (PRS) to predict the molecular subtypes, precision immunotherapy, and prognosis in BLCA.

## Materials and methods

### Data sources

We comprehensively downloaded TCGA-BLCA profiles about gene expression, mutation and clinical data from the UCSC Xena (https://xenabrowser.net/). As for sequencing analysis, we prepared both the count value and the fragments per kilobase per million mapped fragments (FPKM). In particular, we obliterated “invalid data” which lacked sequencing or survival information. Finally, 411 tumor samples were screened from the TCGA-BLCA for further analysis. We captured GSE32894 from the GEO database (https://www.ncbi.nlm.nih.gov/geo/) with the “GEOquery” R package. Our own cohort, the Xiangya cohort (GSE188715) which had a sample size of 56 patients, was developed in our previous study (Li et al., 2021).

### Unsupervised clustering

In total, we digged out 244 platelet-related genes from the study by Xie et al. (2021). We adopted consensus clustering with a thousand repetitions. Platelet-related genes were showed in Supplementary Table S1.

### Pathway enrichment analysis

We comprehensively captured signatures mainly indicating anti-cancer immunity and ICI therapy efficacy from previous studies (Mariathasan et al., 2018; Braun et al., 2020). Moreover, we obtained other therapeutic signatures with corresponding data from the Drugbank, which wasdescribed detailedly in our published article (Hu et al., 2021). In addition, we collected signatures which could indicate the molecular subtypes of BLCA from the existing documents (Kamoun et al., 2020). Subsequently, the sample-level enrichment scores of those signatures were computed.

To sift out differentially expressed genes (DEGs), we performed differential analysis of gene expression and the threshold was set as the absolute log2 fold change (FC) ˃1 with adjusted *p*-value ˂ 0.05. Information about the GO knowledgebase, the KEGG database and sets of hallmark genes were sourced by the MSigDB (http://www.gsea-msigdb.org/gsea/index.jsp) and analyzed using GSEA (Liberzon et al., 2015).

### Tumor immune microenvironment depiction

The previous study concluded the anti-cancer immunity cycle including 7 key steps and displayed the achievements on the tracking tumor immunophenotype (TIP, http://biocc.hrbmu.edu.cn/TIP/), where we downloaded relevant data applied in our own analysis (Li et al., 2021). In addition, to calculate relative abundances of diverse immune cell types, we adopted the ssGSEA algorithm with data from the TCGA-BLCA and our Xiangya cohort based on gene sets reported previously (Charoentong et al., 2017).

### Development and validation of a platelet risk score

We first constructed a univariable Cox regression model to screen prognostic genes from colletced platelet-related genes. Afterwards, we narrowed down the prognostic genes using the least absolute shrinkage and selector operation (LASSO) with ten-fold cross validation. Finally, 13 genes were selected, and the Cox proportional hazard regression was used to generate a PRS, carried out by the “glmnet” R package:

PRS=Σβі * RNAi

We further validated our PRS in external databases including GSE32894 and Xiangya cohort.

### Assessment of molecular subtypes of BLCA

BLCA patients were successfully distinguished by diverse criteria of seven molecular subtypes using “ConsensusMIBC” and “BLCAsubtyping” R packages. In addition, we regrouped these subtypes and renamed them after “basal” and “luminal” subtypes withpublished methods (Kamoun et al., 2020). Previous studies provide a detailed description (Hu et al., 2021; Li et al., 2021).

### Statistical analysis

The differences between normally distributed continuous variables were evaluated with the *t*-test. Furthermore, we performed the Wilcoxon rank-sum test. Categorical values were compared using the χ2 or Fisher’s exact test. The Kaplan-Meier method and log-rank tests were used for survival analysis. Pearson’s correlation coefficients were used to evaluate correlations among the variables. Time-dependent receiver operating characteristic (ROC) analysis was set up to evaluate the predictive accuracy of our model. *p*-values for DEG and GSEA analyses were adjusted using the false discovery rate method. Two-sided *p* values ˂ 0.05 was set as the criterion of significance. The R version in all our analyses was 4.13.

## Result

### Platelet regulated patterns in BLCA

**FIGURE 1:**
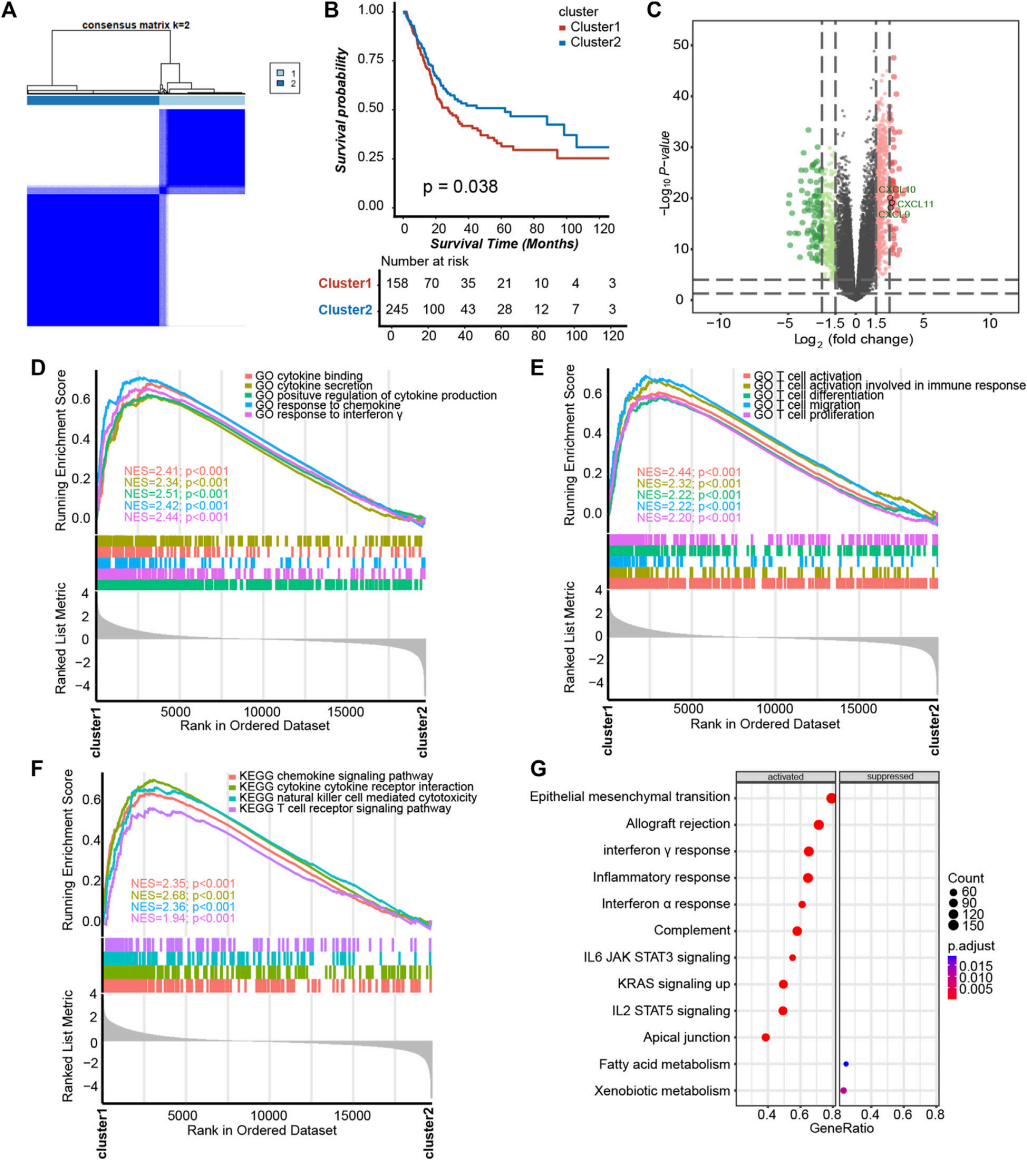
Platelet-regulated patterns in BLCA. **(A)** Unsupervised clustering divided 244 platelet-related genes into two clusters, and **(B)** Kaplan–Meier plots revealed that the survival probability of patients exhibited sensibly higher in platelet cluster2 than cluster1. **(C)** The volcano plot showed DEGs between two platelet clusters. **(D)** Cytokine- and chemokine-related pathways in gene ontology (GO) pathways using gene set enrichment analysis (GSEA) analysis. **(E)** GSEA analysis revealed that platelet cluster1 showed significant enrichment of T cell-related pathways in GO pathways. **(F)** T cell-related pathways, chemokine, cytokine and NK cell in Kyoto Encyclopedia of Genes and Genomes (KEGG) pathways using GSEA analysis. **(G)** GSEA of hallmark pathways between two platelet-regulated patterns.

To determine whether platelet-related genes were comprehensively regulated by a specific pattern in BLCA, we divided patients in TCGA-BLCA into binary clusters, namely, the platelet cluster1 and platelet cluster2 respectively (Figure 1A, Supplementary Figures S1A–SF). Interestingly, survival analysis results showed that patients in platelet cluster2 displayed better survival outcomes than those in platelet cluster1, with *p* = 0.038 (Figure 1B). Surprisingly, a positive correlation was found between immune activation and cluster1. Further, 2,520 DEGs between these two clusters were finally sifted out (Supplementary Table S2), and then used to generate a volcano plot which indicated that some significant chemokines, including CXCL9, CXCL10, and CXCL11, had increased expression levels in platelet cluster1 (Figure 1C). Further analysis showed that pathways related to cytokines and chemokines were activated in cluster1 (Figure 1D, Supplementary Table S3); hence, we wondered if there was more infiltration of effector immune cells in platelet cluster1. As expected, pathways related to T cells and natural killer (NK) cells were obviously activated (Figures 1E, F, Supplementary Table S2). Furthermore, we performed GSEA for hallmark pathways and observed that the majority of these immune-related pathways were activated in platelet cluster1, including epithelial mesenchymal transition, complement, and apical junction (Figure 1G, Supplementary Table S2), suggesting that platelet cluster1 might represent an immune-activated phenotype relative to platelet cluster2.

### Description of immune characteristics between two clusters

**FIGURE 2:**
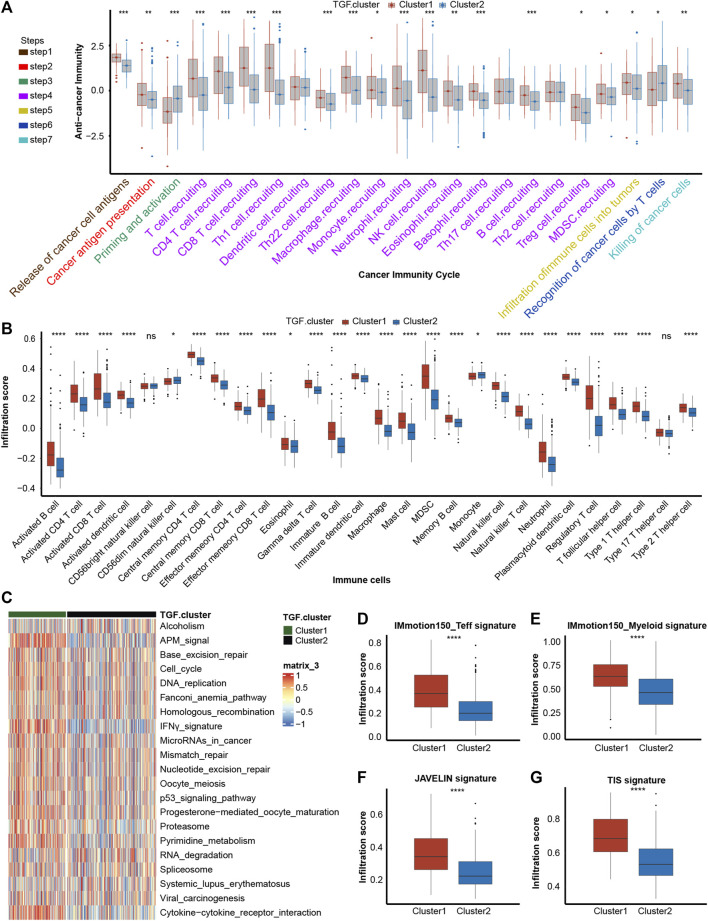
Description of immune characteristics between two clusters. **(A)** Differences in each step of tumor immunity cycle between two platelet-regulated patterns. **(B)** Different immune cells infiltration status between two platelet-regulated patterns. **(C)** The heatmap showed that most of twenty-one pathways contributing to immunotherapy efficacy predicting were sensibly upregulated in platelet cluster1. **(D–G)** Box plots showed the estimation and comparison of four immune-related pathways including IMmotion150 Teff signature **(D)**, IMmotion150 Myeloid signature **(E)**, JAVELIN **(F)** and tumor inflammation signature **(G)** between two platelet regulated patterns. The amount of * from one to four means that the corresponding *p*-value is less than 0.05, 0.01, 0.001, 0.0001, respectively. “ns” means that *p* values more than 0.05 and the result is of no significance.

To determine if platelet cluster1 represented an inflamed TME phenotype and cluster2 represented a non-inflamed phenotype of BLCA, we further identified the differences between these two clusters in cancer immune cycle. Most steps in the cancer immune cycles showed sensibly higher levels in platelet cluster1 than in cluster2, which indicated a greater level of immune activation and immune cell infiltration in the tumor microenvironment in platelet cluster1 (Figure 2A). We performed ssGSEA and observed that a variety of TILs, such as dendritic cells (DCs), macrophages, T cells of different kinds and neutrophils, were significantly higher in platelet cluster1 than in platelet cluster2 (Figure 2B). Overall, these results suggested that platelet cluster1 had an inflamed phenotype that could be responsive to ICI treatment, whereas platelet cluster2 had a non-inflamed phenotype, which could not respond to ICI treatment. A previous study summarized 21 pathways contributing to the prediction of immunotherapy efficacy (Mariathasan et al., 2018), and we observed that almost all these pathways (such as APM signal, cell cycle, DNA replication and so on) were highly regulated in platelet cluster1 (Figure 2C). What’s more, four immune-related signatures were found significantly activated in cluster 1 compared to cluster 2 (Figures 2D–G). These results further support the notion that patients in platelet cluster1 might be responsive to ICI treatment. Unfortunately, the results of unsupervised clustering could hardly explain a single patient’s regulation pattern, as it was conducted based on the population. Therefore, we intended to screen out novel genes and construct a risk score.

### Construction and validation of a platelet risk score and its clinical significance

**FIGURE 3:**
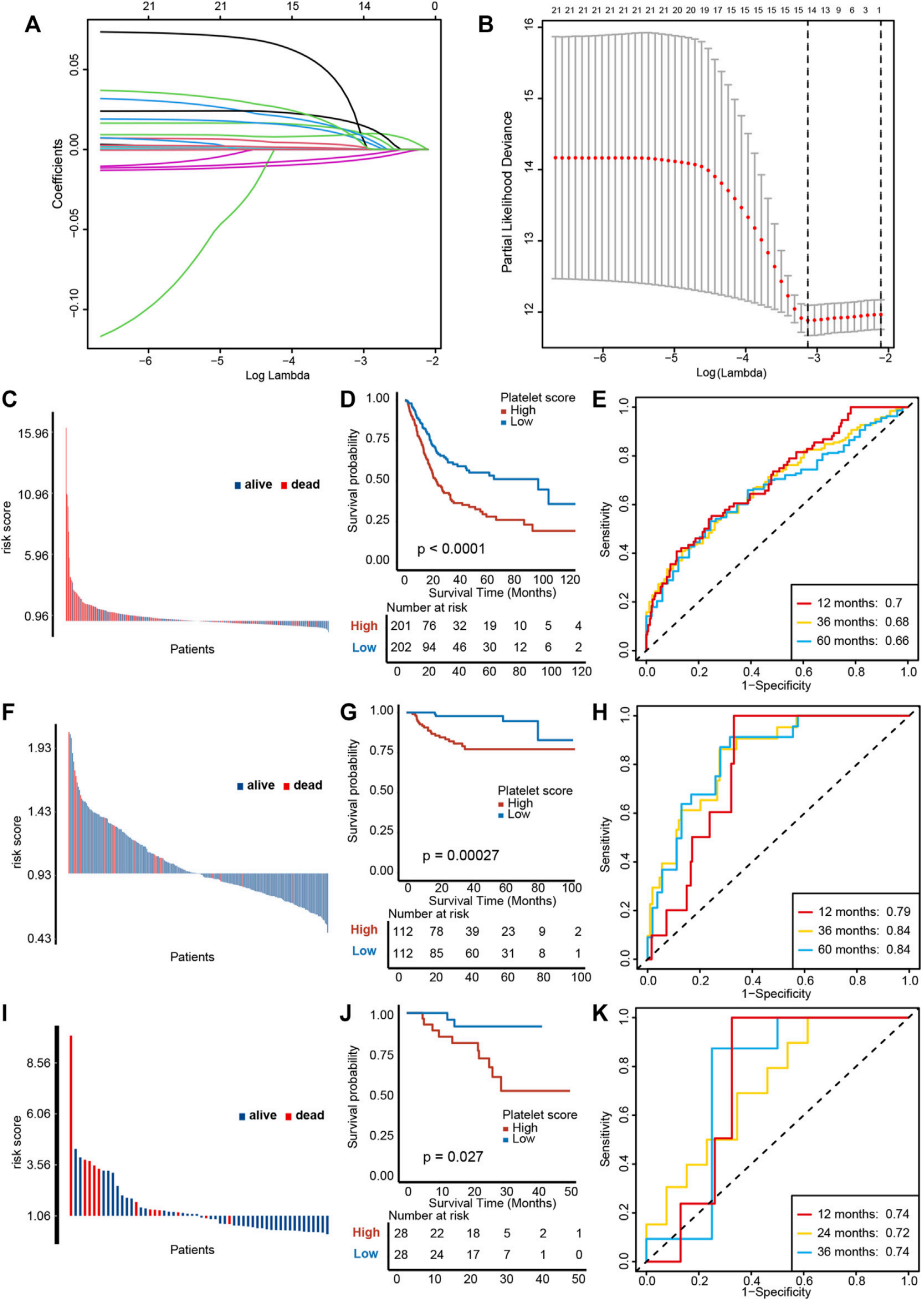
Construction and validation of a platelet risk score (PRS) and its clinical significance. **(A)** Results of LASSO regression based on platelet related genes. **(B)** Results of ten-fold cross validation. **(C)** Distribution of patients’ survival status demonstrated that patients with high PRS in TCGA-BLCA exihibit poorer overall survival outcomes. **(D)** Kaplan-Meier analysis revealed that higher PRS corresponded to particularly poorer survival outcomes in TCGA-BLCA. **(E)** ROC curves of PRS showed satisfactory predictive accuracy of PRS in TCGA-BLCA cohort. **(F–H)** The validation of PRS prognosis value in GSE32894. **(I–K)** The validation of PRS prognosis value in the Xiangya validation cohort.

We performed a univariable Cox regression analysis and LASSO with 10-fold cross validation based on 244 platelet-related genes to screen out 14 genes with prognostic value (Figures 3A, B, Supplementary Table S4). In particular, due to KIF26A lacking expression in all other GEO databases, it was removed from these 14 genes. Based on the remaining 13 genes, we constructed a PRS by the Cox proportional hazard regression algorithm. In TCGA-BLCA training cohort, higher PRS corresponded to particularly poorer survival outcomes (Figures 3C, D, *p* < 0.0001), and an area under the curve (AUC) was approximately 0.68, indicating the accuracy of prognostic prediction was satisfactory (Figure 3E). Then, we used the GSE32894 and Xiangya cohort to validate the prognosis value of PRS. In GSE32894, a higher PRS resulted in poorer survival outcomes (Figures 3F, G, *p* = 0.00027), with satisfactory predictive accuracy (Figure 3H, AUCs around 0.82). In the Xiangya cohort, a higher PRS corresponded to shorter survival times and poorer survival outcomes (Figures 3I, J, *p* = 0.027) and the predictive accuracy was also satisfying (Figure 3K, AUC around 0.73). Taken together, these results suggest that our PRS may predict the individual prognosis of patients with BLCA.

We further performed multivariable Cox regression analysis on age, gender, grade, tumor stage, and PRS. We observed that age, tumor stage and PRS were independent risk factors for overall survival (Supplementary Figure S2A). In order to quantifying individual risk assessment, we constructed a nomogram with these three parameters (Supplementary Figure S2B). Supplementary Figure S2C demonstrates the consistence between actual outcomes and the predictive overall survival outcomes, indicating a high accuracy of our nomogram. The corresponding AUCs at 1, 3, and 5 years were 0.74, 0.71, and 0.70, respectively (Supplementary Figures S2D–SF).

### Platelet risk score for immune cell infiltration evaluation of individual patient

**FIGURE 4:**
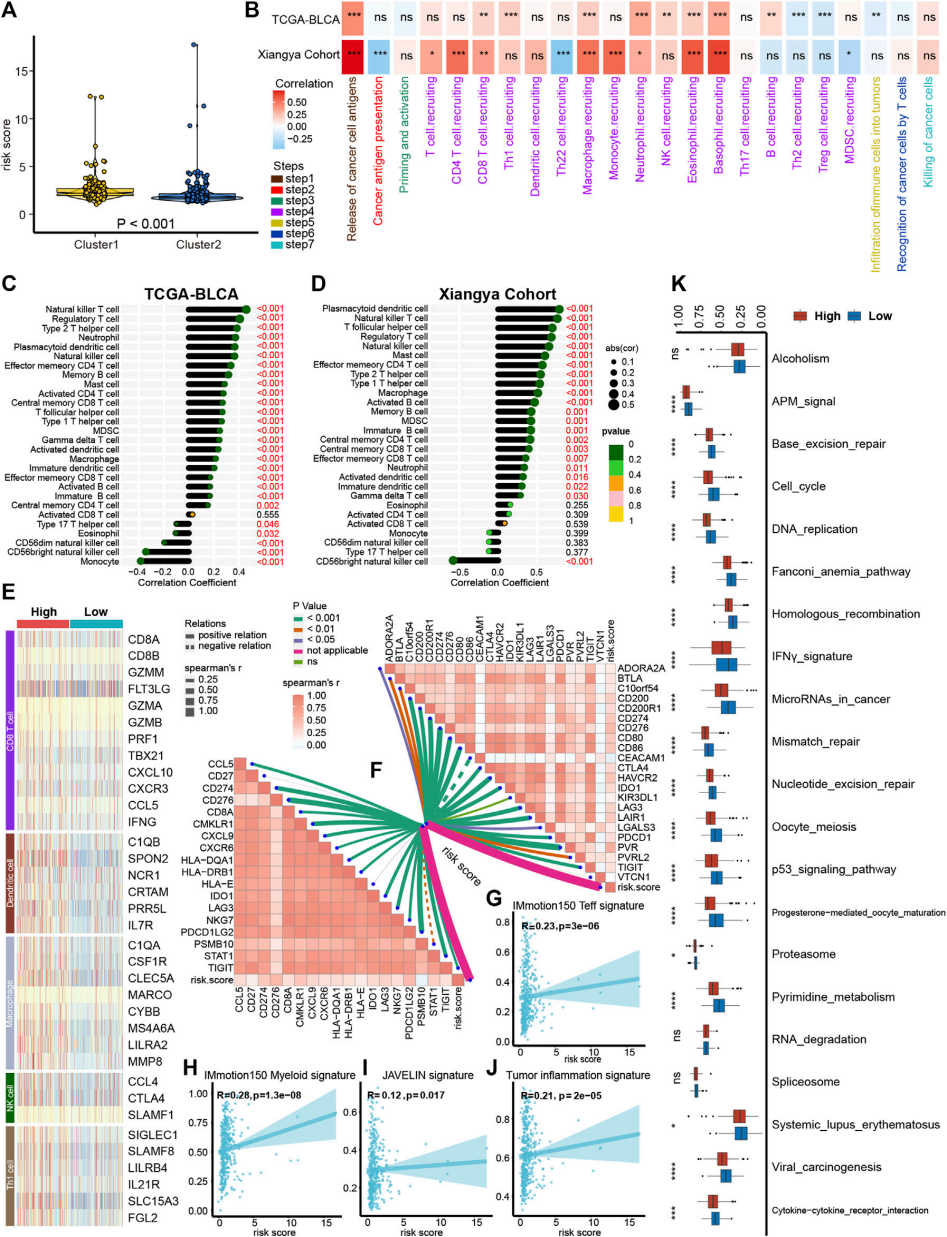
Platelet risk score (PRS) for individual patient’s immune cell infiltration evaluation. **(A)** The connection between platelet cluster 1/2 and PRS. **(B)** The heatmap showed that in both the TCGA-BLCA and Xiangya cohort, PRS had a positive correlation with most steps of cancer immunity cycle. **(C–D)** PRS had a positive correlation with immune cells infiltration in TCGA-BLCA **(C)** and Xiangya cohort **(D)**. **(E)** The effect genes of five types of tumor-infiltrating immune cells were sensibly upregulated in high PRS group. **(F)** Our PRS had a positive correlation with 22 ICI-relevant genes (right upper) and 18 TIS genes (left bottom). **(G,H)** Our PRS had a positive correlation with IMmotion150 T-effector signature **(G)**, IMmotion150 Myeloid signature **(H)**, JAVELIN signature **(I)**, and tumor inflammation signatures **(J)**. **(K)** Different regulation of immunotherapy related pathways in high and low PRS groups. The amount of * from one to four means that the corresponding *p*-value is less than 0.05, 0.01, 0.001, 0.0001, respectively. “ns” means that *p* values more than 0.05 and the result is of no significance.

As demonstrated in Figures 2A, B, platelet cluster1 and cluster2 represent the inflamed and non-inflamed TME phenotypes, respectively. We further analyzed the difference of immune infiltration between platelet cluster 1/2 and PRS and found that platelet cluster1 corresponds to high PRS, while platelet cluster2 corresponds to low PRS (Figure 4A). Therefore, we used the PRS to predict individual TILs infiltration based on the 13 platelet-related genes.

As expected, in the TCGA-BLCA and Xiangya cohort, PRS had a significantly positive correlation with most steps of tumor immune cycle (Figure 4B, Supplementary Table S5). Furthermore, ssGSEA results showed a positive correlation between TILs infiltration and PRS in TCGA-BLCA and Xiangya cohort, including regulatory T cells, type 2 T helper cells, plasmacytoid dendritic cells, and NK cells (Figures 4C, D, Supplementary Table S5). Our previous studies summarized the effector genes of type 1 helper (Th1) cells, CD8^+^ cells, NK cells, DCs, and macrophages (Hu et al., 2021; Li et al., 2021), and these genes were all positively correlated with high PRS (Figure 4E). Moreover, PRS had a positive correlation with 22 ICI-relevant genes including CTLA4, CD80 and CD86, as well as 18 TIS genes including CCL5, CD27 and STAT1 (Figure 4F, Supplementary Table S6). For the prediction of immunity therapy efficacy, our PRS showed a significantly positive correlation with IMmotion150 Teff signature (Figure 4G, R = 0.23, *p* < 0.001), IMmotion150 Myeloid signature (Figure 4H, R = 0.28, *p* < 0.001), JAVELIN signature (Figure 4I, R = 0.12, *p* = 0.017) and TIS (Figure 4J, R = 0.21, *p* < 0.001). Furthermore, a significant upregulation of all 21 immune therapy-related pathways was observed in the high PRS group (Figure 4K).

### PRS for predicting molecular subtypes of BLCA and other therapeutic applications

**FIGURE 5:**
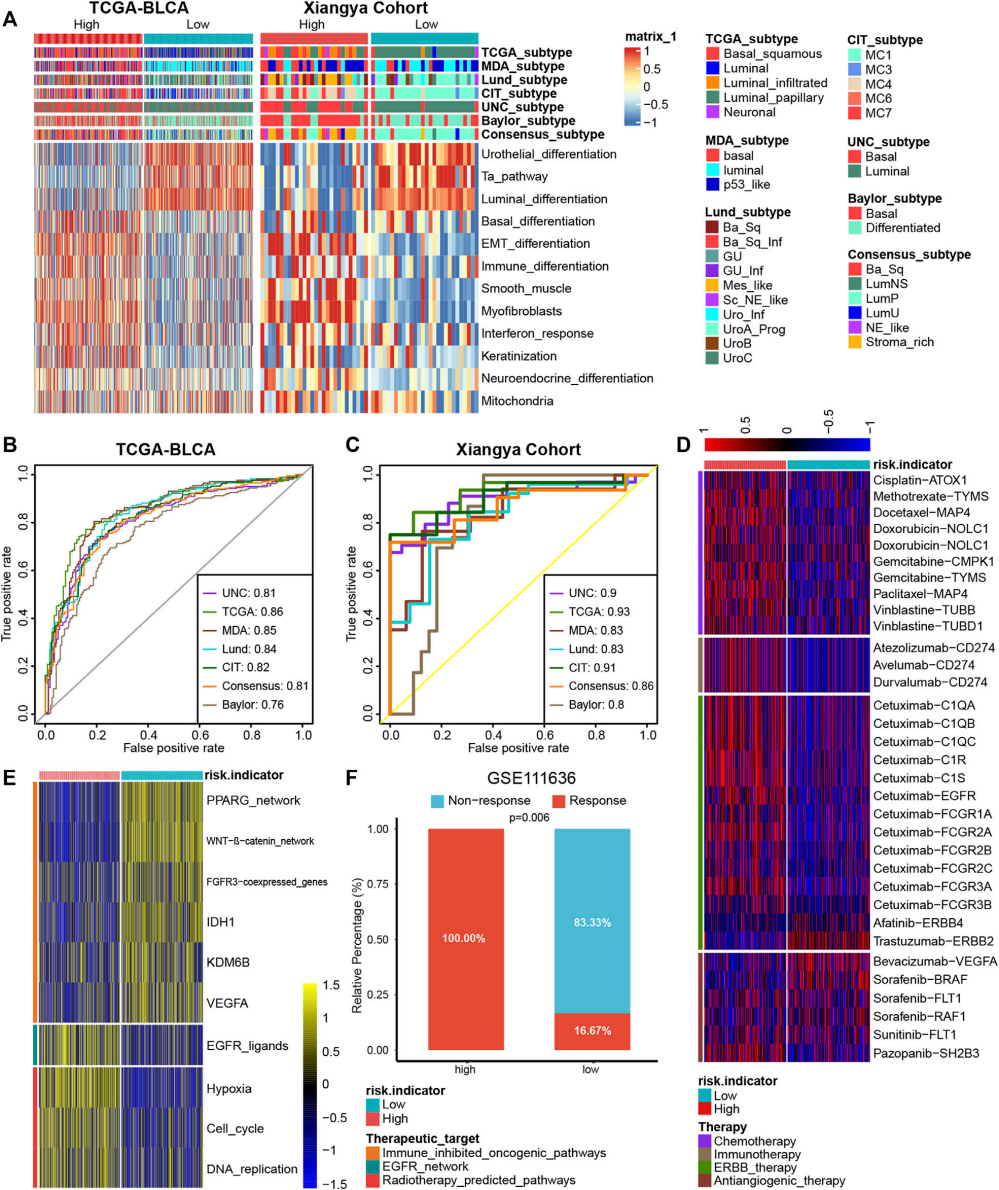
PRS for predicting molecular subtypes of BLCA and other therapeutic opportunities. **(A)** BLCA with high PRS stood for a basal subtype and low PRS stood for a luminal subtype in TCGA-BLCA and Xiangya cohort. **(B,C)** The predictive accuracies of PRS for predicting luminal and basal subtypes were satisfying in both TCGA-BLCA and Xiangya cohort. **(D)** As demonstrated in the heatmap, the high and low PRS group had different response to different therapies, according to the DrugBank database. **(E)** The heatmap demonstrated different regulation of the pathways related to different therapies were differently in high and low PRS groups. **(F)** The proportion of patients in the high/low signature group who respond to anti-PD-1 therapy in GSE111636.

Different molecular subtypes of BLCA exhibit different responsiveness to immunotherapy treatments; a luminal subtype exhibits longer survival time and lower immunotherapy response rates than a basal subtype (Mariathasan et al., 2018; Kamoun et al., 2020). As demonstrated in Figure 5A, BLCA with high PRS stood for a basal subtype enriched for basal differentiation and epithelial-mesenchymal transition (EMT) differentiation pathways, whereas BLCA with low PRS stood for a luminal subtype enriched for urothelial, Ta, and luminal differentiation pathways, which is in accordance with previous results. What’s more, the predictive accuracies of PRS for predicting luminal and basal subtypes were satisfying in both TCGA-BLCA and Xiangya cohort (Figures 5B, C, AUC around 0.85).

In addition to predicting immunotherapy efficacy, our PRS could predict other therapeutic opportunities. The heat map demonstrated that the high PRS group had a significantly higher response to chemotherapy, immunotherapy, and ERBB therapy, whereas the low PRS group had a lower response to antiangiogenic therapy, according to the DrugBank database (Figure 5D). As shown in Figure 5E, immune-inhibited oncogenic pathways were upregulated in the low PRS group, whereas EGFR network and radiotherapy-predicted pathways were upregulated in the high PRS group, which indicated that EGFR and radiotherapy treatment could be more suitable for patients with high PRS and immune-inhibited treatment could bring better outcomes for patients with low PRS. As some immunotherapy cohorts lack survival outcomes, so we constructed platelet signature based 13 genes using ssGSEA algorithm. Then we compared immunotherapy (anti-PD-1/PD-L1) response between high and low signature groups in four immunotherapy cohorts. As expected, in GSE111636 (anti-PD-1), 100% of patients in high signature group showed response to immunotherapy, which was significantly higher than that in low signature group (Figure 5F). In another three cohorts (anti-PD-1, GSE165252 and GSE126044; anti-PD-L1, GSE173839), although without statistical significance, there were more response patients in the high signature group than that in the low signature group (Supplementary Figures S3A–C).

## Discussion

Platelets, a type of significant blood cell contributing to coagulation and hemostasis, were found to influence cancer progression and metastasis in various ways (Haemmerle et al., 2018). Platelets can aggregate on tumor cells and form a physical barrier to prevent NK cell’s attacks (Goubran et al., 2013). Additionally, various growth factors such as TGF-β and PDGF are released by platelets and promote tumor growth by promoting angiogenesis, inducing the expression of tissue factors and other ways (Tesfamariam, 2016; Saito et al., 2018). In BLCA, scientists have found that the platelet-to-lymphocyte ratio has the potential to predict the efficacy of various therapies (Zhang et al., 2015; Wang et al., 2022; Wu et al., 2022). The diagnosis and prognosis predicting values of hemoglobin-to-platelet ratio, platelet-to-leukocyte, platelet volume and platelet distribution width have been also explored in BLCA (Schulz et al., 2017; Tang et al., 2018; Song et al., 2022a). Thus, platelet-related genes are potential targets of cancer therapy. Xie et al. summarized 480 platelet-related genes, the majority of which were reported to take part in tumor development and tumor immune evasion (Xie et al., 2021). Among these genes, α/β-hydrolase domain-containing 6 (AhBHD6) participates in the migration and invasion of non-small cell lung cancer cells (Tang et al., 2020). Type I and II interferons (IFNs) contributes to restricting anti-tumor immunity by CD^+^8 T cell exhaustion (Lukhele et al., 2022). Importantly, the downregulation of the tyrosine kinase Lck/Yes-Related Novel Protein (LYN) was reported to prevent breast tumor invasion and metastasis, and ICI treatment combined with anti-LYN therapy might improve the efficacy of ICIs (Fattet et al., 2020; Vlachostergios, 2021). However, few studies have comprehensively analyzed the relationship between platelet-related genes, cancer immunity, and immunotherapy.

Recently, lots of studies concentrating on a single gene revealed the novel mechanism of TME in BLCA, such as ETV4 and HSF1 (Huang et al., 2022; Zhang et al., 2023). Factually, the relationship between the TME and a group of genes has been also extensively studies. Previously, we used 14 cuproptosis-related genes to generate cuproptosis clusters and observed the correlation between those clusters with TME and immunotherapy in BLCA (Li et al., 2022). Zou et al. generated two programmed cell death clusters, and then comprehensively studied the relationship between those two clusters and prognosis and drug sensitivity in triple-negative breast cancer (Zou et al., 2022). Chen et al. identified three pyroptosis modification patterns and their TME immune characteristics in response to three immune phenotypes (Liu et al., 2022). In BLCA, Song et al. divided patients into four distinct iron metabolism patterns that play vital roles in TME regulation (Song et al., 2022b). These studies could uncover the immune evasion mechanism and are beneficial to precise immunotherapy for patients with BLCA. However, no study has systematically correlated platelet-related genes with the TME, prognosis, and immunotherapy efficacy in BLCA. In this research, we generated two platelet clusters with different survival outcomes, TME phenotypes, and immunotherapy sensitivity. Furthermore, we generated a PRS for individual prognosis and tumor immune infiltration prediction.

BLCA has become the 10th most common malignancy worldwide and poses a huge threat to public health (Sung et al., 2021). Approximately 75% of BLCA cases are non-muscle invasive bladder cancers (NMIBC) with a high recurrence rate (Berdik, 2017; Babjuk et al., 2019). Survival outcomes and response to neoadjuvant chemotherapy are worse when NMIBC develops into muscle invasive bladder cancer (MIBC) (Pietzak et al., 2019). Because BLCA is an immunogenic cancer, ICIs show promising survival benefits in some patients (Sharma et al., 2017; Patel et al., 2020). A clinical trial first found that atezolizumab exhibited good tolerability and durable activity in advanced BLCA. Furthermore, this study was the first to correlate TCGA subtypes with responses to immune checkpoint inhibition (Rosenberg et al., 2016). In addition, nivolumab and pembrolizumab were demonstrated to improve the survival outcomes in advanced BLCA (Sharma et al., 2016; Bellmunt et al., 2017). Nevertheless, we observed that not all patients in these researches were sensitive to ICI treatment, which necessitates the use of biomarkers for predicting ICI efficacy.

Recent studies have found that tumors cannot be separated from the low immune surveillance and drug interference provided by the TME (Binnewies et al., 2018; Crispen and Kusmartsev, 2020). More TILs and an immuno-activated microenvironment can enhance the efficacy of ICI treatment in patients with BLCA (Zou et al., 2021). Thus, discriminating inflamed tumors from non-inflamed tumors could contribute to predicting ICI efficacy, and the transformation of non-inflamed tumors into inflamed ones leads to higher ICI efficacy (Duan et al., 2020). Various studies show the correlation of platelets with prognosis and immunotherapy efficacy in colorectal cancer, triple-negative breast cancer, and other cancer types (Xie et al., 2021; Miao et al., 2022). In the present research, we first constructed a PRS for precise prediction of TME phenotypes, and further validated the result in our cohort. In addition, our PRS could directly predict immunotherapy efficacy and other therapeutic opportunities, which are vital for the treatment of patients with BLCA.

The present research had a few limitations. Firstly, prospective studies are needed to compensate for the limitations of our retrospective data. Secondly, further wet experiments should be performed to investigate the underlying mechanisms in detail. Third, batch effects need to be taken into consideration to validate our findings.

## Conclusion

Platelet-related genes predicted TME phenotypes in BLCA. We constructed a PRS that could predict the TME, prognosis, immunotherapy efficacy, and molecular subtypes in BLCA.

## Data Availability

The original contributions presented in the study are included in the article/Supplementary Material, further inquiries can be directed to the corresponding authors.

## Supplementary material

SUPPLEMENTARY FIGURE S1 Unsupervised clustering generally divided 244 platelet-related genes in the TCGA-BLCA cohort into two clusters.

SUPPLEMENTARY FIGURE S2 Construction of a nomogram. **(A)** Multivariable Cox regression analysis indicated three independent risk factors for overall survival PRS and other clinicopathological factors. **(B)** Details of the nomogram. **(C)** Calibration curves showed the consistence between actual outcomes and the predictive overall survival outcomes. **(D–F)** The corresponding AUCs at 1 **(D)**, 3 **(E)**, and 5 **(F)** years were 0.74, 0.71, and 0.70, respectively.

SUPPLEMENTARY FIGURE S3 The proportion of patients in the high/low signature group who respond to anti-PD-1/PD-L1 therapy in GSE165252 **(A)**, GSE126044 **(B)** and GSE173839 **(C)**, respectively.
