# Supplementary figures and images for: A novel platelet risk score for stratifing the tumor immunophenotypes, treatment responses and prognosis in bladder carcinoma: results from real-world cohorts
Source: Front Pharmacol. 2023 May 4;14:1187700. doi: 10.3389/fphar.2023.1187700 (PMC10192868; doi:10.3389/fphar.2023.1187700)

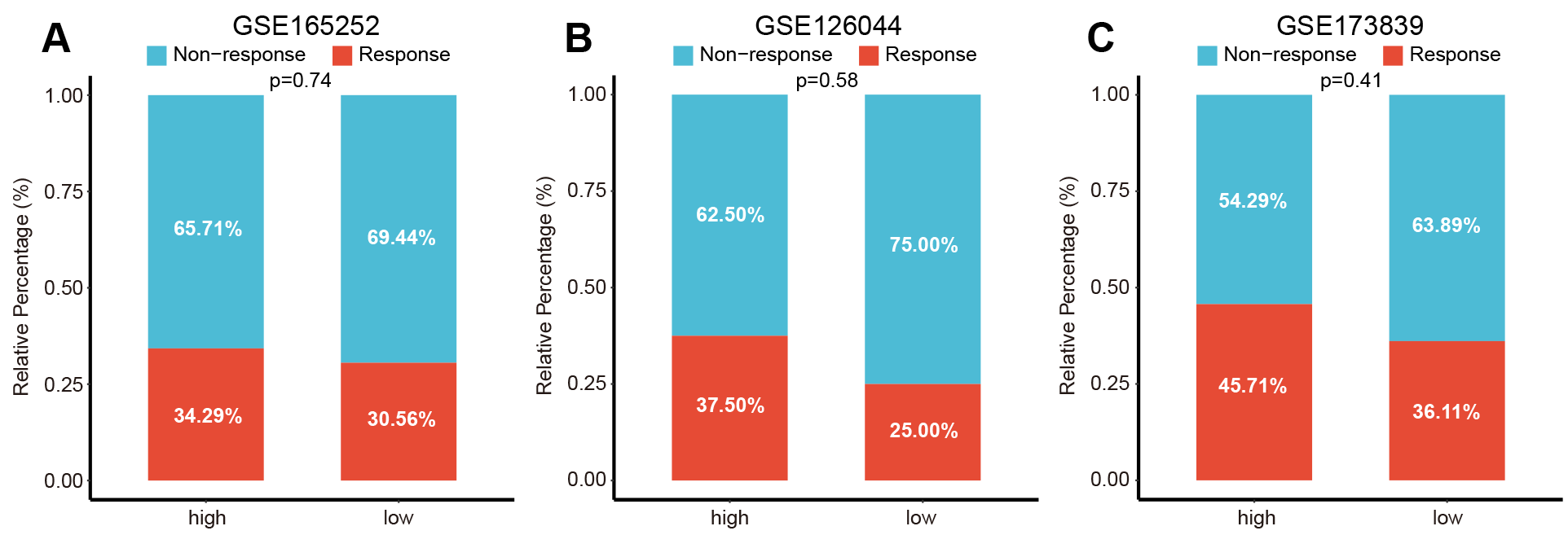

Supplement: Supplementary file 3 [file Image3.TIF]

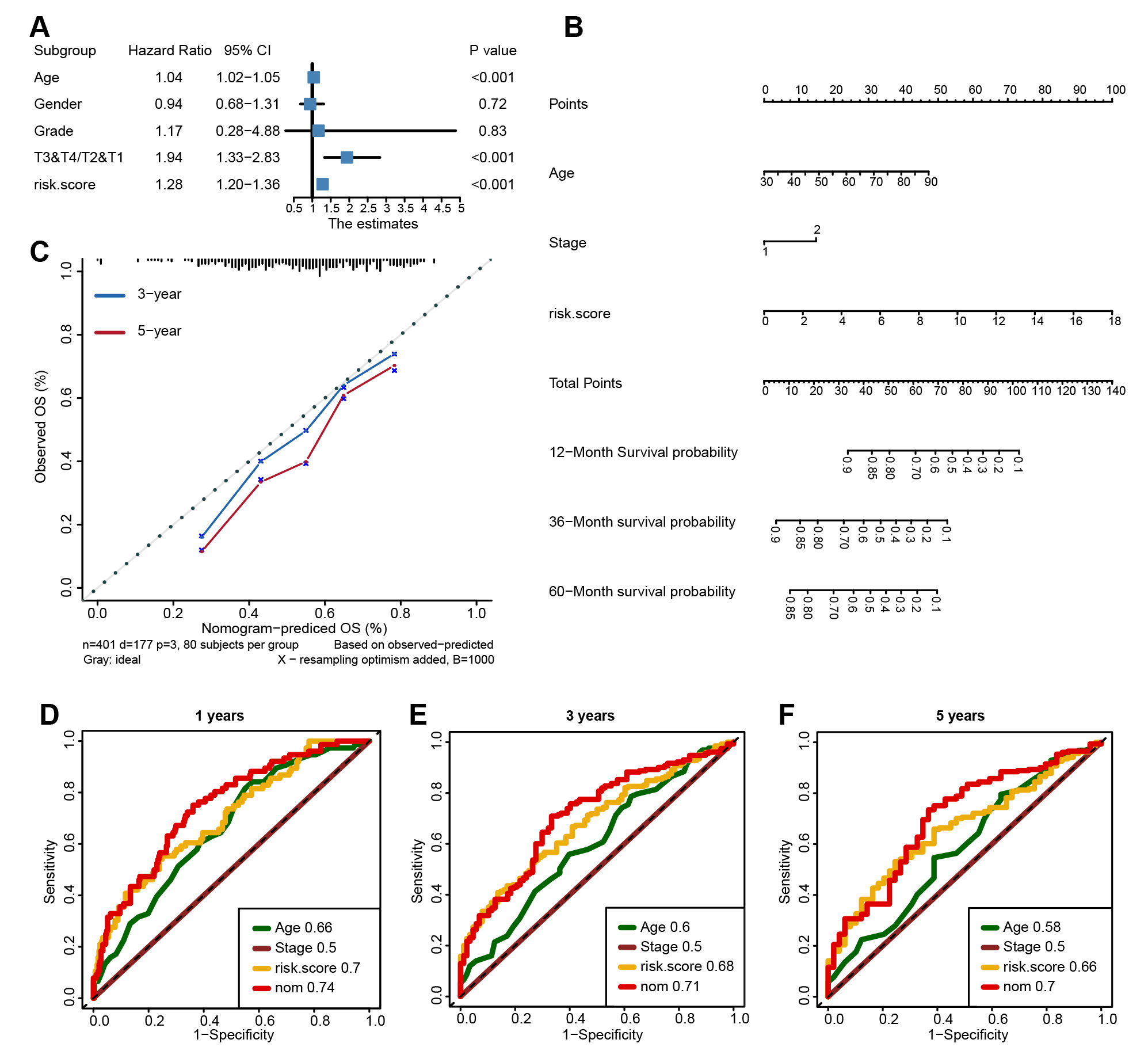

Supplement: Supplementary file 4 [file Image2.TIF]

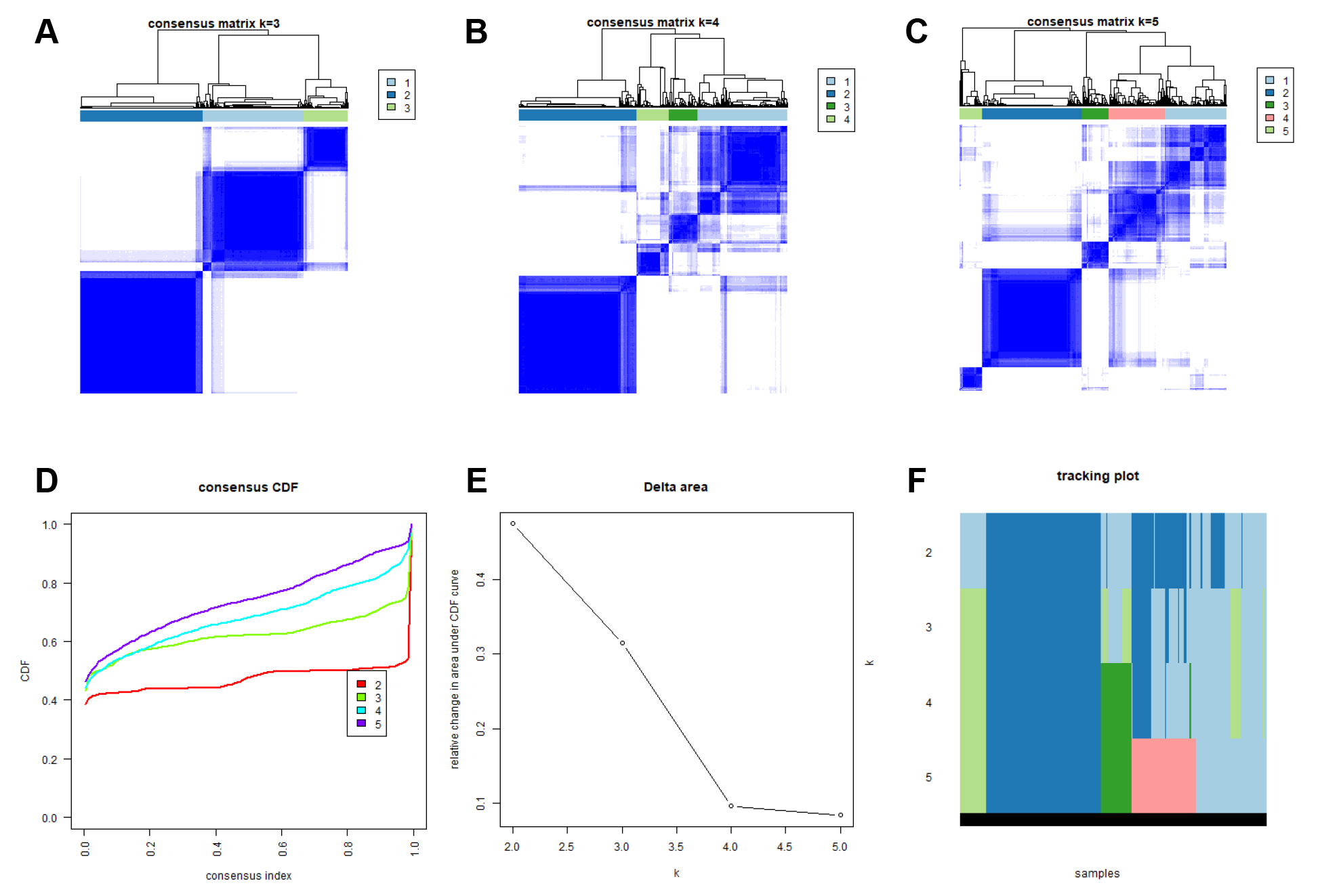

Supplement: Supplementary file 5 [file Image1.TIF]
